# Would large dataset sample size unveil the potential of deep neural networks for improved genome-enabled prediction of complex traits? The case for body weight in broilers

**DOI:** 10.1186/s12864-020-07181-x

**Published:** 2020-11-09

**Authors:** Tiago L. Passafaro, Fernando B. Lopes, João R. R. Dórea, Mark Craven, Vivian Breen, Rachel J. Hawken, Guilherme J. M. Rosa

**Affiliations:** 1 Department of Animal and Dairy Sciences, University of Wisconsin, Madison, WI 53706 USA; 2 Cobb-Vantress Inc., Siloam Springs, AR 72761 USA; 3 Department of Biostatistics & Medical Informatics, University of Wisconsin, Madison, WI 53706 USA; 4 Department of Computer Sciences, University of Wisconsin, Madison, WI 53706 USA

**Keywords:** Body weight, Broilers, Deep neural networks, Genome-enabled prediction, And multilayer perceptron

## Abstract

**Background:**

Deep neural networks (DNN) are a particular case of artificial neural networks (ANN) composed by multiple hidden layers, and have recently gained attention in genome-enabled prediction of complex traits. Yet, few studies in genome-enabled prediction have assessed the performance of DNN compared to traditional regression models. Strikingly, no clear superiority of DNN has been reported so far, and results seem highly dependent on the species and traits of application. Nevertheless, the relatively small datasets used in previous studies, most with fewer than 5000 observations may have precluded the full potential of DNN. Therefore, the objective of this study was to investigate the impact of the dataset sample size on the performance of DNN compared to Bayesian regression models for genome-enable prediction of body weight in broilers by sub-sampling 63,526 observations of the training set.

**Results:**

Predictive performance of DNN improved as sample size increased, reaching a plateau at about 0.32 of prediction correlation when 60% of the entire training set size was used (i.e., 39,510 observations). Interestingly, DNN showed superior prediction correlation using up to 3% of training set, but poorer prediction correlation after that compared to Bayesian Ridge Regression (BRR) and Bayes Cπ. Regardless of the amount of data used to train the predictive machines, DNN displayed the lowest mean square error of prediction compared to all other approaches. The predictive bias was lower for DNN compared to Bayesian models, across all dataset sizes, with estimates close to one with larger sample sizes.

**Conclusions:**

DNN had worse prediction correlation compared to BRR and Bayes Cπ, but improved mean square error of prediction and bias relative to both Bayesian models for genome-enabled prediction of body weight in broilers. Such findings, highlights advantages and disadvantages between predictive approaches depending on the criterion used for comparison. Furthermore, the inclusion of more data per se is not a guarantee for the DNN to outperform the Bayesian regression methods commonly used for genome-enabled prediction. Nonetheless, further analysis is necessary to detect scenarios where DNN can clearly outperform Bayesian benchmark models.

## Background

The identification and selection of individuals with superior genetic merit is critical for the improvement of complex traits in animals and plants. Genomic selection was originally proposed by Meuwissen et al. (2001) [1], and been used as a tool to accelerate the genetic improvement of complex traits by earlier and accurate selection of genetically superior individuals compared to traditional pedigree analysis [2, 3]. Advances in genotyping technologies allowed the production of high-density genetic chips in a cost-effective manner, making genomic selection a reality for animal [4–6] and plant [7, 8] breeding programs.

Genomic selection relies on the information of a large number of genetic markers, posing a statistical challenge for genome-enabled prediction studies in which the number of markers is often much larger than the number of observations. Methods such as G-BLUP [9], Bayes A and Bayes B [1], Bayes C [10], Bayesian Lasso [11], Single-step analysis [12], among others have been proposed to cope with this challenge and also to improve the performance of genome-enabled prediction. In addition, machine learning (ML) techniques have also been implemented in genome-enabled prediction in attempt to improve predictive performance due to their ability to accommodate nonlinear relationships between predictors and response variables. ML methods such as the Reproducing Kernel Hilbert Space [13, 14], Random Forest [15], and Artificial Neural Networks (ANN) [16, 17] have been used in genome-enabled prediction, showing slightly better or similar results compared to linear regression approaches. Recently, a particular case of ANN with multiple hidden layers, namely, Deep Neural Networks (DNN) has emerged as one of the most powerful machines for pattern recognition, being successfully applied in different fields such as bioinformatics and computer vision. Applications in the former field include the investigation of the regulatory role in DNA-binding protein [18, 19], estimation of the effects of non-coding sequence variants [20, 21], and improve DNA sequencing analysis [22], while the latter field applications include image recognition [23, 24] and object detection [25].

Deep neural networks are gaining prominence also in genome-enabled prediction and they have been already employed in different studies [26–30]. However, results reported by these studies have shown no clear superiority of DNN compared to traditional linear regression approaches, with results seem highly dependent on species and traits of application. Nevertheless, the relatively small datasets used in previous studies, most with fewer than 5000 observations may have precluded the full potential of DNN. For the most successful applications of DNN, the dataset sample sizes had at least 70,000 observations (e.g., MNIST, ImageNet, and VoxCeleb). Thus, large sample sizes could be crucial to unveil the potential of DNN in the genome-enabled field. Bellot et al. (2018) [29] employed DNN for genome-enabled prediction of complex traits in humans using a large dataset composed of 102,221 observations, finding similar performance of DNN and Bayesian regression models. Hence the question remains if DNN cannot indeed out-perform Bayesian regression models commonly used in genome-enable prediction of complex traits, or if its performance depends on the species and trait being considered, or if there is also a dependence on the dataset sample size used for training the models. Here we try to tackle this latter enquiry, by assessing the relative performance of DNN with varying sizes of training sets. Specifically, we employ a sub-sampling scheme from a large dataset sample of broiler chickens, and compare the results from DNN and Bayesian regression models on genome-enabled prediction of body weight of broilers.

## Results

### Genetic parameter estimates

Estimates of variance components for body weight were 4436.6 (SE = 281.07), 1026.0 (SE = 71.12), and 13,477.0 (SE = 163.01) g^2^ for additive genetic, maternal permanent environmental, and residual effects, respectively. These estimates resulted in a phenotypic variance of 18,939.6 (SE = 146.59) g^2^. Estimate of direct heritability for body weight was 0.23 (SE = 0.013), and the proportion of the phenotypic variance due to maternal permanent environmental effect was 0.05 (SE = 0.003).

### Deep neural networks architecture search

**Fig. 1:**
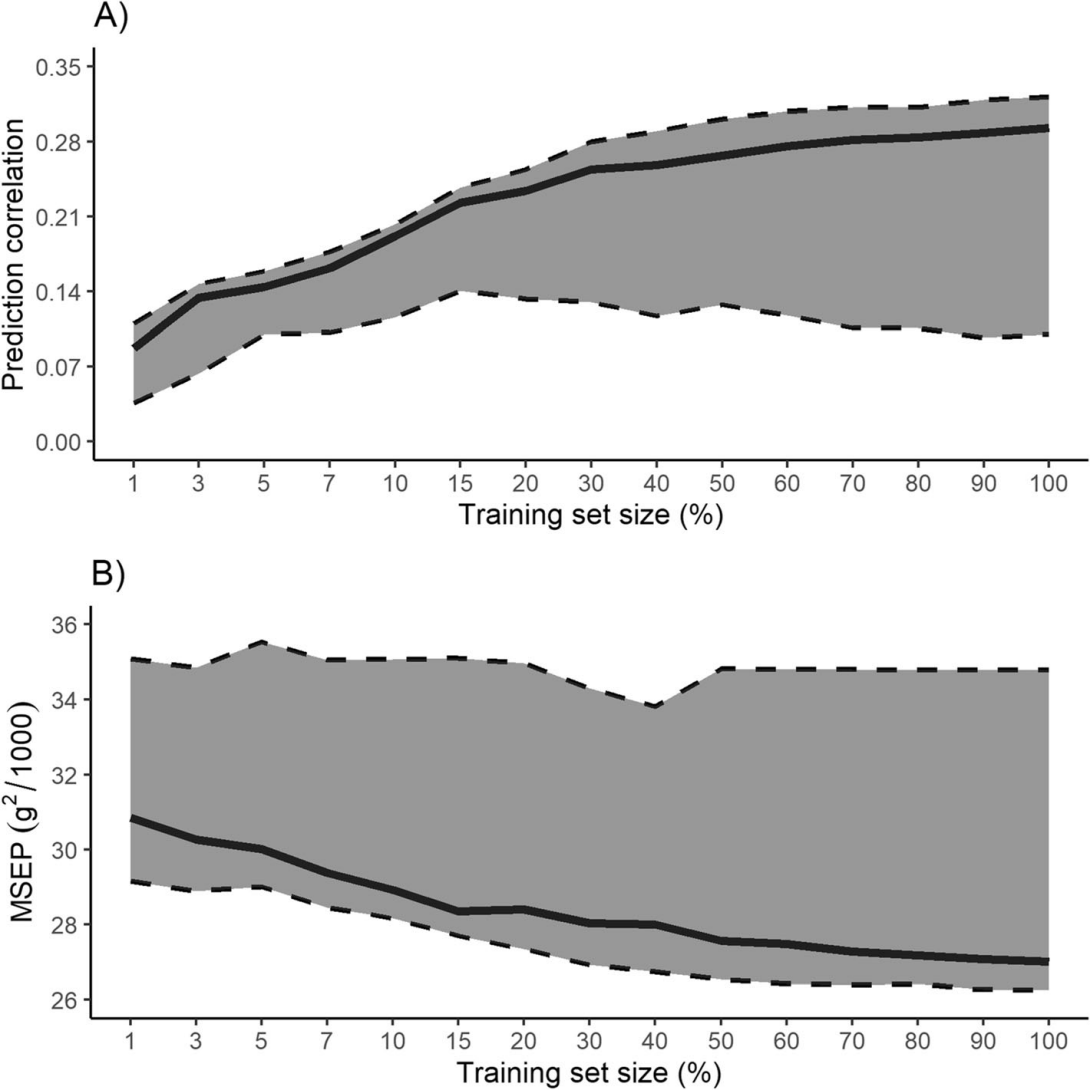
Predictive performance for each of the 200 deep neural networks generated using the neural architecture search, in (**a**) prediction correlation and (**b**) mean square error of prediction (MSEP). The continuous black line represents the median of the 200 deep neural networks for each sub-sample of the training set

The DNN architecture search was performed based on the “random search” described in Goodfellow et al. (2016) [31]. The searching process considered 200 different DNN architectures that were tested for each sub-samplings of the training set, considering the hyperparameters space provided in Table 1. Deep neural networks were selected based on their prediction correlation on the tuning set. Different architectures of the DNN were selected for each sub-sampling of the training set (Table 2). Overall, DNN with more than one hidden layer showed a greater predictive performance considering up to 50% of the training set size, while simple ANN architectures with one hidden layer and approximately 300–800 units were selected afterwards. All ANN had a L2 norm (ridge regularization) larger than zero, and the dropout rate was smaller than 1, except for the models using 1, 3, and 100% of the entire training set size. The prediction correlation of all ANN is summarized in Fig. 1a. Regardless of the ANN architecture, the prediction correlation had an increased trend with larger sample sizes. Interestingly, the distance between the worst to the median prediction correlation of all ANN was greater than the distance between the best to the median prediction correlation of all ANN for each sub-sample of the training set. The MSEP for each ANN are summarized in Fig. 1b. Overall, the MSEP had a decreased trend with larger sub-samples sizes. Similarly, to the prediction correlation, the distance between the worst to the median MSEP of all ANN was greater than the distance between the best and the median MSEP of all ANN for each sub-sample of the training set.

**Table 1:** Hyperparameters considered in the neural architecture search of deep neural networks (DNN)^a^

Hyperparameter	Space
Number of units	1, 100, 200, 300, 400, 500, 600, 700, 800, 900, 1000, 2000, 3000, 4000, 5000
Hidden layers	1, 2, 3, 4
Dropout rate^b^	0.5, 0.6, 0.7, 0.8, 0.9, 1
L2^c^	0.0000, 0.0025, 0.0050, 0.0075, 0.0100, 0.0125, 0.0150, 0.0175, 0.0200, 0.0225, 0.0250, 0.0275, 0.3000, 0.0325, 0.0350, 0.0375, 0.0400, 0.0425, 0.0450, 0.0475, 0.0500, 0.0525, 0.0550, 0.0575, 0.0600, 0.0625, 0.0650, 0.0675, 0.0700, 0.0725, 0.0750, 0.0775, 0.0800, 0.0825, 0.0850, 0.0875, 0.0900, 0.0925, 0.0950, 0.0975, 0.1000

^a^The hyperparameters were randomly select and combined to find the optimal DNN architecture

^b^The dropout rate was applied in all layers, except for the output layer

^c^L2 = ridge regularization

**Table 2:** The best deep neural network architecture selected based on prediction correlation on the tuning set for each sub-sampling of the training set

Size (%)	Deep neural network architecture					
	Number of layers	Number of units per layer^a^	L2^b^	Dropout rate^c^	Accuracy	MSEP^d^
1	4	5000^(1)^-1^(2)^-600^(3)^-800^(4)^	0.0600	1.0	0.090	30,589.3
3	4	5000^(1)^-300^(2)^-200^(3)^-4000^(4)^	0.0675	1.0	0.137	29,649.9
5	3	400^(1)^-200^(2)^ -900^(3)^	0.0100	0.5	0.145	30,408.7
7	2	500^(1)^-2000^(2)^	0.0450	0.8	0.166	29,062.4
10	2	800^(1)^-100^(2)^	0.0025	0.6	0.200	28,440.9
15	2	800^(1)^-900^(2)^	0.0050	0.5	0.236	27,755.0
20	4	600^(1)^-100^(2)^-500^(3)^-700^(4)^	0.0325	0.5	0.226	28,849.5
30	1	1000^(1)^	0.0100	0.7	0.274	27,025.5
40	1	2000^(1)^	0.0800	0.6	0.285	26,877.4
50	3	600^(1)^-4000^(2)^ -100^(3)^	0.0975	0.5	0.285	27,250.3
60	1	300^(1)^	0.0800	0.8	0.304	26,622.3
70	1	400^(1)^	0.0800	0.5	0.309	26,506.4
80	1	800^(1)^	0.0925	0.7	0.308	26,484.5
90	1	400^(1)^	0.0800	0.5	0.307	26,710.1
100	1	500^(1)^	0.0600	1.0	0.322	26,264.8

^a^The number in parenthesis represents the corresponding hidden layer

^b^L2 = ridge regularization

^c^Dropout rate was applied in all layers, except for the output layer

^d^MSEP = mean square error of prediction

### Models’ predictive performance

**Fig. 2:**
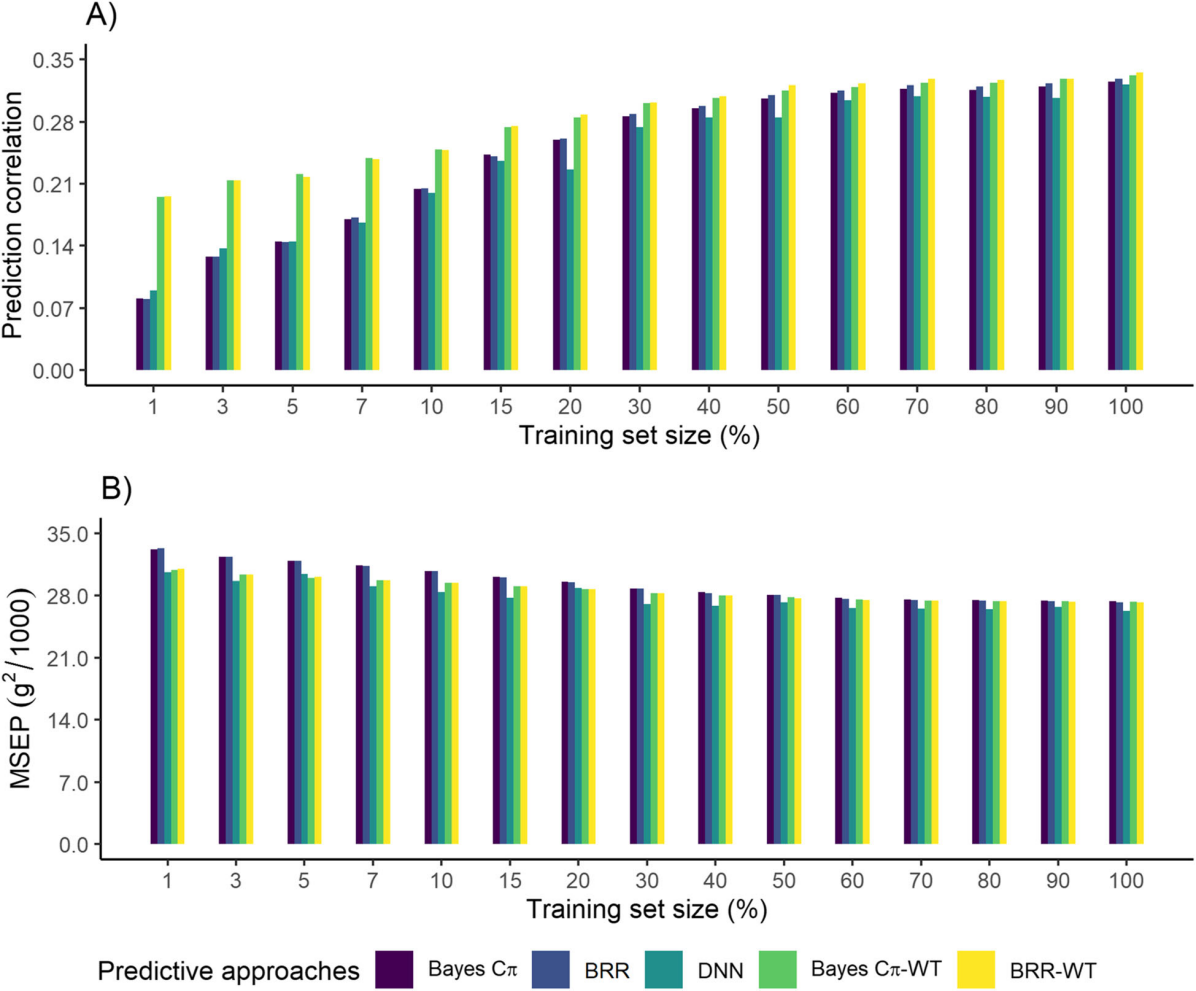
Predictive performance for Bayes Cπ, Bayesian Ridge Regression (BRR), Deep Neural Networks (DNN), Bayes Cπ fit with the tuning set (Bayes Cπ-WT), and Bayesian Ridge Regression fit with the tuning set (BRR-WT) for each sub-sampling of the entire training set size, in (**a**) prediction correlation and (**b**) mean square error of prediction (MSEP)

As expected, the prediction correlation increased with larger training sample sizes, with a fast increment using up to 50% of the available data, reaching a plateau of approximately 0.32 afterwards, for each genome-enabled prediction approach (Fig. 2a). Deep neural networks had the greatest prediction correlation using 1% (0.090) and 3% (0.137) of the training set size, while Bayesian Ridge Regression (BRR) and Bayes Cπ fit without the tuning set showed similar or better prediction correlation compared to DNN when more than 5% of the entire training set size was considered. The relative gain of prediction correlation for DNN compared to BRR (Bayes Cπ) was 11% (13%) and 7% (7%), when 1 and 3% of the entire training set size was used, respectively; it was however worse afterwards, varying from − 13% to − 1%. After fitting the Bayesian regression models with the additional data from the tuning set in each sub-sampling of the training set, the prediction correlation of Bayesian Ridge Regression (BRR-WT) and Bayes Cπ (Bayes Cπ-WT) were greater than the DNN, regardless of the amount of data used. Moreover, the relative gain of DNN compared to BRR-WT (Bayes Cπ-WT) decreased remarkably to − 116% (− 117%) and − 56% (− 56%) using 1 and 3% of the training set size, respectively, but such difference in the relative gain was attenuated with larger sample sizes. Overall, the MSEP decreased along with the sample size of the training set for all predictive approaches (Fig. 2b). Deep neural networks showed the lowest mean square error of prediction (MSEP) for each subset of the training set, ranging from 26,264.8 to 30,589.3. The relative gain of MSEP was better for DNN compared to BRR (Bayes Cπ), ranging from − 2% (− 2%) to − 8% (− 8%) when 20% (20%) and 3% (3%) of the entire training set size was used, respectively. Interestingly, the MSEP of BRR-WT and Bayes Cπ-WT were greater than DNN for each sub-sampling of the training set, except when 20% of the training data was used.

**Fig. 3:**
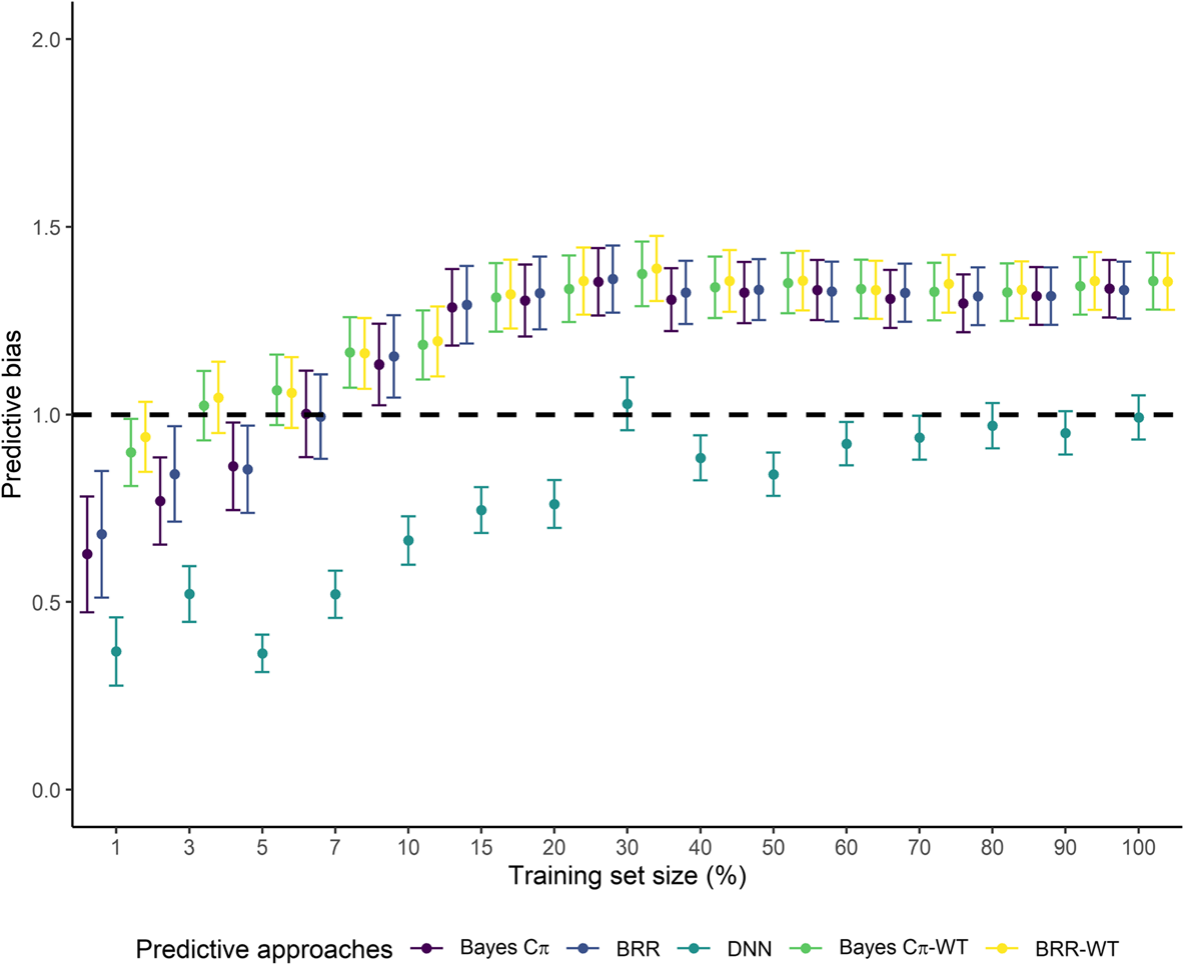
Predictive bias for Bayes Cπ, Bayesian Ridge Regression (BRR), Deep Neural Networks (DNN), Bayes Cπ fit with the tuning set (Bayes Cπ-WT), and Bayesian Ridge Regression fit with the tuning set (BRR-WT) for each sub-sampling of the entire training set size

Deep neural networks showed the smallest predictive bias compared to all Bayesian regression models (Fig. 3). Interestingly, the predictive bias of DNN was smaller than one for all partitions of the training set, except when using 30% of the data in the training set. Conversely, Bayesian regression models had a predictive bias greater than one for almost all training set sub-samples, starting after the sub-sampling of 10 and 5% of the training set for models fit with or without the tuning set, respectively. Spearman rank correlations for body weight prediction in broilers varied from 0.32 to 0.99 between genome-enabled prediction approaches under different training set sample sizes (Table 3). Correlations were higher between Bayesian models, and lower between DNN and Bayesian models. The agreement on the top 10-ranked broilers selected across the genome-enabled prediction approaches ranged from 26 to 96% under different training set sample sizes (Table 4). Similar to the prediction correlation, estimates were higher between BRR and Bayes Cπ, and lower between both of them and the DNN.

**Table 3:** Spearman rank correlations between predicted body weight from the different genome-enabled prediction approaches and for the various sub-sampling of the entire training dataset

Predictive approach	Training dataset size (%)														
	1	3	5	7	10	15	20	30	40	50	60	70	80	90	100
BRR x Bayes Cπ	0.99	0.99	0.99	0.99	0.99	0.99	0.99	0.99	0.98	0.98	0.98	0.98	0.98	0.98	0.98
BRR x DNN	0.79	0.89	0.86	0.95	0.95	0.96	0.78	0.97	0.94	0.91	0.94	0.95	0.95	0.95	0.94
BRR x BRR-WT	0.33	0.52	0.64	0.69	0.78	0.83	0.87	0.91	0.94	0.95	0.96	0.96	0.96	0.97	0.97
BRR x Bayes Cπ-WT	0.33	0.52	0.63	0.69	0.79	0.83	0.85	0.90	0.93	0.93	0.95	0.95	0.95	0.96	0.96
Bayes Cπ x DNN	0.79	0.89	0.86	0.96	0.95	0.95	0.78	0.95	0.93	0.88	0.93	0.94	0.94	0.93	0.94
Bayes Cπ x BRR-WT	0.32	0.52	0.64	0.69	0.78	0.82	0.86	0.90	0.93	0.93	0.94	0.95	0.95	0.95	0.96
Bayes Cπ x Bayes Cπ-WT	0.32	0.52	0.63	0.69	0.80	0.82	0.85	0.90	0.93	0.94	0.95	0.95	0.96	0.97	0.97
DNN x BRR-WT	0.33	0.52	0.58	0.66	0.76	0.79	0.71	0.88	0.89	0.87	0.92	0.92	0.93	0.92	0.92
DNN x Bayes Cπ-WT	0.33	0.52	0.57	0.66	0.76	0.79	0.69	0.87	0.87	0.86	0.90	0.91	0.92	0.91	0.91
BRR-WT x Bayes Cπ-WT	0.99	0.99	0.99	0.99	0.99	0.99	0.98	0.98	0.98	0.97	0.99	0.98	0.98	0.98	0.98

**Table 4:** Agreement on the top 10-ranked broilers selected across the different genome-enabled prediction approaches and for the various sub-sampling of the entire training dataset

Predictive Approach	Training dataset size (%)														
	1	3	5	7	10	15	20	30	40	50	60	70	80	90	100
BRR x Bayes Cπ	91.5	94.5	95.0	94.4	95.9	93.9	93.2	90.8	90.3	87.6	86.6	89.1	87.6	87.9	88.6
BRR x DNN	57.8	66.9	64.9	79.3	78.4	82.6	55.5	79.8	76.6	73.1	72.9	77.1	78.5	75.9	76.5
BRR x BRR-WT	28.2	37.3	44.5	50.1	57.7	62.8	68.5	74.3	78.8	81.5	83.3	84.6	86.4	86.1	87.9
BRR x Bayes Cπ-WT	28.3	36.8	44.9	50.5	58.1	63.4	64.7	72.1	75.8	76.4	79.5	80.1	80.6	83.0	83.5
Bayes Cπ x DNN	57.6	65.7	64.2	78.8	77.9	80.9	55.3	76.9	74.8	69.4	71.6	74.9	75.2	72.2	74.2
Bayes Cπ x BRR-WT	28.5	37.3	44.0	50.7	56.4	62.5	67.6	73.3	76.8	79.1	79.8	82.2	82.0	81.8	82.6
Bayes Cπ x Bayes Cπ-WT	28.7	36.9	44.2	50.9	57.3	63.4	64.1	73.2	75.8	78.7	80.4	81.3	81.3	83.3	85.2
DNN x BRR-WT	28.2	37.5	38.0	47.3	55.3	58.8	47.4	66.5	68.5	68.3	69.7	72.8	73.6	72.2	72.3
DNN x Bayes Cπ-WT	28.1	36.5	38.1	47.4	55.4	58.8	46.3	64.4	67.8	65.2	67.7	71.0	71.0	71.2	70.1
BRR-WT x Bayes Cπ-WT	94.1	96.2	93.3	93.9	94.5	93.9	89.5	88.9	88.7	85.7	89.6	88.6	88.1	88.3	88.2

## Discussion

The heritability estimated for body weight in broiler chickens from a pure line population was of moderate magnitude, accounting for 23% of the phenotypic variance. This result indicates that the response to selection should be effective in a short to medium term. The ratio of maternal permanent environmental variance over the phenotypic variance was low and contributed to 5% of the body weight variation. Although the variance fraction accounted for by the maternal permanent environment effect was relatively low, the inclusion of this effect in the model is essential to avoid an inflation of the variance of the additive genetic effect. Body weight estimates of heritability and the fraction for maternal permanent environmental variance were consistent with other studies using the same trait in broilers from single pure lines [32, 33].

For the DNN implementation, the architecture search was performed by selecting the hyperparameters at random, leading to the selection of different models for each subset of the training set. This result indicates that the choice of the best DNN architecture was strongly affected by the amount of data available during training. Therefore, the neural network architecture search did not provide a robust DNN structure to predict body weight throughout the training set partitions, representing a disadvantage for DNN compared to the Bayesian regression models. Recently, simulated annealing and genetic algorithms have been considered for hyperparameter optimization in machine learning applications [34, 35]. Such approaches may provide a more robust DNN architecture. However, Bellot et al. (2018) [29] evaluated the performance of DNN on the genome-enable prediction of complex traits in humans using a genetic algorithm for hyperparameter optimization, and also reported that DNN had similar results with Bayesian regression models.

Hyperparameter optimization is a very difficult task, which involves the exploration of various DNN architectures to find an optimal parameter set within a specific search space. Such component of the learning process is crucial for the success of DNN and depends on the definition of the search space, as well as computational resources and time. The optimal search space is unknown and many hyperparameters should be tested to find the best DNN architecture. It is worth mentioning that caution should be taken when defining the search space. Deep neural network architectures with a single unit in the hidden layer may result in extreme information compression, which can adversely affect predictive performance. In our study, 20 out of 200 neural networks architectures selected had at least one hidden layer with a single unit. From these 20 architectures, only one was able to display the best predictive performance, namely when using 1% of the entire training set size. This result indicates that such DNNs may indeed suffer from extreme information compression, as discussed in Szegedy et al. (2014) [36]. The parallel computing as employed in our study can be used to alleviate time issues, where each DNN architecture is trained and evaluated independently on different computers. However, parallel computing requires expensive computational resources, which in most situations is not available for many researchers. Despite such challenges, hyperparameter optimization is critical to obtain DNN architectures which could deliver greater predictive performance. For instance, in our study, the difference of predictive performance between the best and worst DNN in each sub-sampling of the training set was considerably large. Therefore, implementing DNN with no hyperparameter optimization may inadvertently define a DNN architecture that delivers a poor predictive performance. Moreover, the hyperparameter optimization cost is relatively minor compared to the cost to collect, store, and analyze genomic data. Therefore, hyperparameter optimization should be considered for genome-enabled prediction applications in animal and plant breeding programs.

The best models selected for each partition of the training set have some type of regularization (i.e. L2 > 0 and dropout rate < 1) to improve model generalization. The large number of inputs typically observed in genome-enabled prediction, and the high correlation between markers due to linkage disequilibrium may negatively affect the performance of DNN. Regularization approaches such as dropout can prevent complex co-adaptations between units [36], reducing the observed association among inputs from adjacent layers. Therefore, this result suggests that DNN with regularization techniques are recommended to improve predictive performance on new observations for genome-enabled prediction. A similar result was reported by McDowell (2016) [26], who found better predictive performance for DNN with some kind of regularization compared to DNN without regularization for genome-enabled prediction of complex traits in different plant species.

The selection of DNN hyperparameters considering the predictive performance on a tuning set may not reflect the best predictive performance in the testing set. For instance, for each sub-sampling of the training set at least one DNN with different architecture had a greater predictive performance on the testing set compared to those DNN selected based on the lowest MSEP observed in the tuning set. Therefore, selecting DNN architecture by measuring the predictive performance on a tuning set may not deliver optimized predictive performance on new records. Nevertheless, DNN optimization based on the predictive performance on a testing set provides results that are optimistically biased since some information from the testing set is considered a priori. Thus, in our study the correct strategy was to select the DNN architecture based on the predictive performance in the tuning set.

Deep neural networks are gaining prominence in genome-enabled prediction because of several advantages including flexibility to accommodate complex relationships between output variables and predictors, their high predictive performance, and no parametric assumptions regarding variable distributions [37]. Although DNN has emerged with an enormous potential to transform genome-enable prediction, recent studies showed no evident superiority of DNN relative to traditional genome-enable prediction models. For instance, Rachmatia et al. (2017) [27] used deep belief networks to predict complex traits in maize and found that DNN outperformed linear regression models in only 2 out of 8 traits. McDowell (2016) [26] compared DNN with 5 linear regression methods (i.e. ordinary least squares, lasso, ridge regression, elastic net, and Bayesian ridge regression) on 6 traits from 3 different species (i.e. Arabidopsis, maize, and wheat). In this study DNN outperformed traditional regression methods in about 50% of the time. In another study, Montesinos-Lopez et al. (2018) [28] compared a multi-task DNN with Bayesian multi-trait and multi-environment model using complex traits in maize and wheat under different environments. The authors reported a greater predictive performance of DNN when genotype x environmental interactions were not included in the analysis and a lower performance when such terms were considered in the analysis. Bellot et al. (2018) [29] compared convolution (CNN) and multiple layer perceptron (MLP) neural networks with Bayesian regression models in the evaluation of five traits in human. These authors reported no remarkable difference in the predictive performance between Bayesian regression methods and DNN, regardless of the DNN architecture used for genome-enabled prediction. Similarly, Abdollahi-Arpanahi et al. (2020) [30] reported lower predictive performance of CNN and MLP compared to G-BLUP and Bayes B for genome-enabled prediction of conception rate in Holstein. However, those authors found that CNN and MLP had superior predictive performance compared to G-BLUP and Bayes B when using simulated data with large amount of non-additive effects and sample size. According to these studies, the performance of DNN is strongly affected by many factors including the genetic architecture of a trait, the presence of non-additive effects, hyperparameter optimization, and the DNN architecture (e.g., MLP, CNN, and multi-task neural networks) considered for genome-enabled prediction. These findings are consistent with our study, in which Bayesian regression models showed similar or greater prediction correlation than DNN, but worst MSEP.

The lowest MSEP of DNN reflects the predictive bias estimates in each sub-sampling of the training set. Deep neural networks showed greater inflation on the prediction of body weights compared to all Bayesian models using up to 20% of the data, and less biased estimates afterwards, indicating an advantage for DNN over Bayesian models. The Spearman’s correlation and the agreement on the top 10-ranked broilers suggested a re-ranking of animals depending upon to the model used. Such difference in the ranking of broilers is more pronounced between Bayesian regression models fitted with the tuning set in comparison to the other genome-enabled prediction approaches, whereas DNN presented a slightly lower re-ranking of broilers relative to BRR and Bayes Cπ.

Interestingly enough, the predictive performance of DNN was better than the BRR and Bayes Cπ when considering small sample sizes. This result is most likely because of the benefit of using in the training process a tuning set exclusive for DNN. However, after re-fitting the Bayesian regression models including also the tuning set data, such an advantage was accounted for and the superiority of DNN vanished. Strategies such as a k-fold cross-validation within the training set could be considered to select DNN architectures. However, in our study, implementing such an approach was extremely difficult due to the computational cost of performing a k-fold cross-validation in such a big data together with the sub-sampling process in the training set for each genome-enabled prediction approach.

Although DNN often show a greater predictive performance when trained with large sample size, for genome-enable prediction it seems that adding more data per se is not a guarantee to outperform benchmark models. The relative simple nature of the marker inputs (i.e. three genotypes coded as 0, 1 or 2) and the complex essence of quantitative traits may pose a challenge for DNN applied to genome-enabled prediction compared to other successful applications, such as in computer vision [29]. As pointed out by these authors, inputs used in computer vision are more complex and less structured than those available for genome-enabled prediction. Furthermore, the attribute (expected value of trait or genetic risk) used in genome-enabled prediction is often not directly observed, rather it is a function of genetic and environmental factors [29]. Therefore, the characteristics of the response variable and inputs may explain in part the similar predictive performance of DNN and Bayesian methods using large amount of data. Furthermore, body weight inheritance is suggestive to be mainly accounted for by genetic additive effects, with a lower contribution of non-additive genetic effects. Abdollahi-Arpanahi et al. (2016) [38] concluded that the dominance effects had a minor contribution in the phenotypic variation of body weight relative to additive effects. Additive inheritance is often well fitted by traditional linear models used for genome-enabled prediction. On the other hand, ANN is better suited to capture nonlinear relationships by using multiple layers and nonlinear activation functions. For instance, Dórea et al. (2018) [39] reported greater predictive performance of ANN compared to Partial Least Squares on the prediction of dry matter intake in lactating dairy cows, concluding that such a superiority is possibly explained by the ability of ANN to accommodate nonlinear relationships. Therefore, the additive genetic nature of body weight may be another potential explanation for the similar predictive performance between DNN and Bayesian models. Moreover, it is important to mention that the influence of sample size on the predictive performance of DNN compared to other traditional genome-enabled prediction models needs to be investigated in other species and traits of interest. For genome-enabled prediction of body weight in broilers, our results did not show any additional benefit of adding more data to train DNN relatively to traditional models. In fact, the results may have even highlighted the superiority of DNN with smaller sample sizes.

It is important to point out some disadvantages of DNN when applied to genome-enable prediction compared to traditional linear regression models. The first drawback has been previously discussed, and reflects the importance of hyperparameter optimization in DNN performance. The second disadvantage is the lack of biological interpretability of the results obtained with DNN. For instance, extracting information from multiple hidden layers is very difficult, turning the algorithm into a “black box” regarding biological interpretation. A practical example of this lack of interpretability is that the effect of each marker cannot be estimated separately, while SNP effects are easily obtained in traditional linear models used for genome-enabled prediction. Another issue of DNN is that such a predictive approach is more susceptible to overfitting than linear models. In our study, we used early stopping, dropout, and a L2 norm to tackle overfitting and the results indeed suggested that such approaches helped to improve generalization. Despite all of these limitations, DNN had a better performance in terms of MSEP but worst prediction correlation compared to the Bayesian regression models. Therefore, DNN should be more explored in genome-enable prediction to find scenarios in which DNN is clearly superior. Common DNN strategies used in the field of computer science including multi-task DNN (i.e. similar to multi-trait analysis), novel algorithms for parameter optimization, and different types of network structures (e.g. convolution and multi-input networks) can be easily adapted and implemented for further analysis in genome-enabled prediction.

## Conclusions

Results have shown that the prediction correlation of DNN was comparable to Bayesian regression models with larger training set sizes, while DNN had the lowest MSEP. The inclusion of more data in the training set per se is not a guarantee for DNN to outperform traditional linear regression models in genome-enabled prediction applications. Overall, the use of DNN for genome-enable prediction is promising but further research investigating novel algorithms for hyperparameter optimization, multi-trait analysis, and other DNN structures are fundamental to evaluate scenarios where DNN can clearly outperform benchmark models.

## Methods

### Phenotypic and genomic information

The dataset was provided by Cobb-Vantress Inc. (Siloam Springs, AR), and included 79,367 body weight observations (mean = 2141.8 g and SD = 238.17 g), measured at 6 weeks of age on broilers from a single purebred line. The total number of birds in the pedigree was 342,383, with 680 sires and 6216 dams. All broilers recorded for body weight were genotyped with a 60 k single nucleotide polymorphism (SNP) panel. The genotype quality control was performed using the *PLINK* software [40] with markers excluded based on the following criteria: 1) Located at non-autosome chromosomes; 2) Unknown map position; 3) Minor allele frequency (MAF) < 0.01; 4) Call rate < 95%; and 5) Departures from Hardy-Weinberg equilibrium with *P* < 10^− 10^. Subsequently, missing genotypes were imputed using the software *FImpute* [41]. Body weight observations with ±3.5 standard deviations away from the average of their contemporary group were treated as outliers and removed from the analysis. After these editing procedures, 77,476 broilers and 49,362 SNPs were retained for genome-enabled prediction analysis.

### Genome-enabled prediction analysis

Genome-enabled prediction of body weight in broilers was performed in two steps. In the first step, variance components were estimated using the AIRemlf90 software [42], and body weight was pre-adjusted by fitting the following linear mixed model:

$$ \mathbf{y}=\mathbf{X}\boldsymbol{\uptheta } +\mathbf{Zu}+\mathbf{Wc}+\mathbf{e} $$

where **y** is a vector of body weights; **θ** is a vector of fixed effects; **u** and **c** are vectors of random additive genetic and maternal permanent environmental effects, respectively; **X**, **Z**, and **W** are known incidence matrices of fixed, additive genetic, and maternal permanent environmental effects, respectively; and **e** is the vector of residuals. Fixed effects in the model were sex (2 levels) and contemporary groups (500 levels). Random effects were assumed to be independent from each other with distributions $\mathbf{u}\sim \mathrm{N}\left(0,\mathbf{A}{\upsigma}_{\mathrm{u}}^2\right)$, $\mathbf{c}\sim \mathrm{N}\left(0,\mathbf{I}{\upsigma}_{\mathrm{c}}^2\right)$, and $\mathbf{e}\sim \mathrm{N}\left(0,\mathbf{I}{\upsigma}_{\mathrm{e}}^2\right)$, where **A** is the additive genetic relationship matrix (342,383 × 342,383), **I** is an identity matrix of appropriate order (i.e. 333 × 333 for maternal permanent environmental and 77,476 × 77,476 for residuals), and ${\sigma}_u^2$, ${\sigma}_c^2$, and ${\sigma}_e^2$ are the variance components for additive genetic, maternal permanent environmental and residual effects, respectively. The pre-adjusted body weight was defined as ${\mathbf{y}}^{\ast}=\mathbf{y}-\mathbf{X}\hat{\boldsymbol{\uptheta}}-\mathbf{W}\hat{\mathbf{c}}$. In the second step, the pre-adjusted body weight was fit on the genetic markers by using three predictive approaches: BRR, Bayes Cπ, and DNN.

### Bayesian regression models

The Bayesian regression analyses were implemented with the R package *BGLR* [43]*.* The Markov Chain Monte Carlo sampling process was performed for each Bayesian model considering a chain of 30,000 iterations, from which the first 20,000 cycles were discarded as a warm-up period, keeping posterior samples for every 5 iterations. The general statistical model for BRR and Bayes Cπ was:

$$ {\mathbf{y}}^{\ast}=\mathbf{1}\mu +\sum \limits_{j=1}^k{\mathbf{z}}_{\mathbf{j}}{a}_j+\mathbf{e} $$

where **y**^**∗**^ is the n × 1 vector of pre-adjusted body weights; *μ* is an overall intercept; *k* is the number of markers; **z**_*j*_ is a n × 1 vector denoting the genotypes of the animals for marker *j*; *a*_*j*_ is the effect of marker *j*, and **e** is the vector of residuals. SNP genotypes were standardized to display mean 0 and variance 1. The vector of residuals **e** was assumed to follow a Gaussian distribution with mean zero and variance ${\sigma}_e^2$.

### Bayesian Ridge Regression

In BRR, an independent prior Gaussian distribution with mean zero and variance ${\upsigma}_a^2$ was assigned to each SNP, so that $\mathrm{p}\left({a}_1,{a}_2,\dots, {a}_k|{\upsigma}_a^2\right)={\prod}_{j=1}^{\mathrm{k}}\mathrm{N}\left({a}_j|0,{\upsigma}_a^2\right)$. The unknown variance parameter ${\sigma}_a^2$ is the same for all genetic markers, and a scaled inverse chi-squared distribution ${X}^{-2}\left({v}_a,{\mathrm{S}}_a^2\right)$ was specified as a prior distribution, where ${\mathrm{S}}_a^2$ and *v*_*a*_ are a scale parameter and degrees of freedom, respectively. A flat prior distribution was assigned for the overall constant *μ*, and similarly to ${\sigma}_a^2$, a scaled inverse chi-square distribution ${X}^{-2}\left({v}_e,{\mathrm{S}}_e^2\right)$ was defined as the prior distribution for the residual variance ${\sigma}_e^2$.

### Bayes Cπ

The Bayes Cπ method was proposed by Habier et al. (2011) [10], and assumed that the vast majority of genetic markers have null effect, with a small proportion of SNPs with non-null effect. The prior distribution of the genetic markers (*a*_*j*_) depends on the variance ${\upsigma}_{a_j}^2$ and the probability π of a marker having zero effect. These mixture of distributions are described as follows:

$$ {a}_j\mid \pi, {\sigma}_a^2=\left\{\begin{array}{cc}0& \mathrm{with}\ \mathrm{probability}\ \pi \\ {}\sim \mathrm{N}\left(0,{\sigma}_a^2\right)& \mathrm{with}\ \mathrm{probability}\ \left(1-\pi \right)\end{array}\right. $$

A scale inverse chi-square distribution with parameters *v*_*a*_ and ${S}_a^2$ was assumed for ${\upsigma}_a^2$, in which *v*_*a*_ = 4.2 and ${S}_a^2=\frac{{\overset{\sim }{\sigma}}_{\mathrm{a}}^2\left({v}_a-2\right)}{v_a}$. The parameter ${\overset{\sim }{\sigma}}_{\mathrm{a}}^2$ is equal to $\frac{{\overset{\sim }{\sigma}}_{\mathrm{s}}^2}{\left(1-\uppi \right){\sum}_{\mathrm{j}=1}^{\mathrm{k}}{p}_j\left(1-{p}_j\right)}$, where ${\overset{\sim }{\sigma}}_s^2$ is the variance explained by all markers and *p*_*j*_ is the allele frequency of the *j*^*th*^ SNP. In the Bayes Cπ method, π is treated as unknown and a uniform *prior* distribution (0,1) was assigned to this parameter. The inclusion/exclusion of each marker in the model is modeled by an indicator variable *δ*_*j*_, which is equal to 1 if the marker *j* is fitted into the model, and zero otherwise. The prior distribution for the overall constant *μ* and the residual variance ${\sigma}_e^2$ were the same as those specified in the BRR model.

### Deep neural networks

**Fig. 4:**
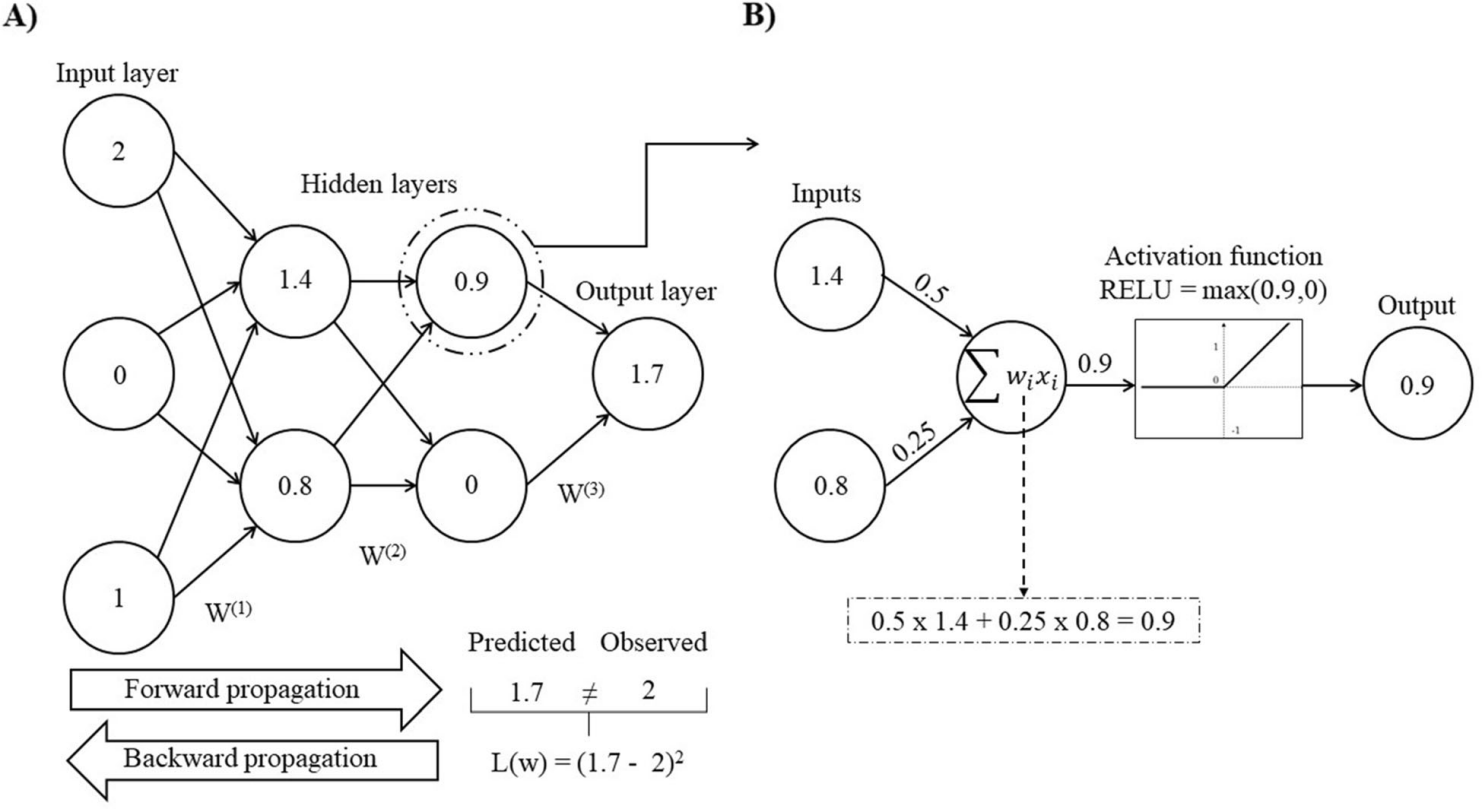
Representation of a Multilayer Perceptron (MLPs) architecture. In (**a**) The structure of the deep neural network (DNN) and the training process including forward and backward propagation are depicted. In the forward propagation information flows from the input to the output layers by outputting the calculations of the activation function to the next layer. In the backward propagation, the output is assessed and a loss function L(W) [i.e. mean square error] is used to minimize the overall error function, and consequently update the network weights using stochastic gradient descent. In (**b**) The underlying calculations for each unit in order to provide the output to the next layer. In this process, weight vectors [W^(.)^] and inputs are linearly combined and transformed based on an activation function, i.e., rectified linear which outputs the maximum between zero and the linear combination of weights and inputs. This figure is based and adapted from the diagram proposed by Angermueller et al. (2016) [44]

Deep neural networks were implemented using a Multilayer Perceptron (MLPs) architecture, in which units from adjacent layers are fully connected, and contained a minimum of 3 layers (Fig. 4). The first layer was the input layer composed by 49,361 units, with each unit representing an SNP. Initially, the inputs for each unit were coded as 0, 1, and 2, representing the number of copies for the reference allele. The inputs were standardized (mean = 0, variance = 1) to avoid numerical issues during the training of DNN. In this setting, each unit (i.e., SNP) has similar interpretation of a covariate in a linear regression model. The last layer in the MPLs is called output layer and provides the prediction (output) of the pre-adjusted body weight. The layers between input and output layers are defined as hidden layers. Connections between units are established through a structure referred to as weights. The training process of DNN occurs in two steps: forward and backward propagations. In the forward propagation, the information flows from the input to the output layer, and each unit transforms a linear combination of weights and inputs from the previous layer with an activation function, and outputs it to the units in the next layer. The output of each unit is obtained as ${o}_r={g}_t\left({\sum}_{s= 1}^z{w}_{rs}{x}_s\right)$, where *g*_*t*_(.) is the activation function, *w*_*rs*_ is the weight of the *r*^*th*^ unit to *x*_*s*_ input of neuron s from the previous layer, and *z* is the number of units in the previous layer. The information propagates in a similar way through the DNN layers, reaching the output layer that provides the prediction for pre-adjusted body weight. A linear activation function, i.e. ${\sum}_{s=1}^z{w}_{rs}{x}_s$, was applied to the output layer, while rectified linear activation functions, i.e. $\max \left({\sum}_{s=1}^z{w}_{rs}{x}_s,0\right)$, were considered for neurons in the hidden layers. In the backward propagation step, the weights of the DNN are adjusted via a stochastic gradient descent algorithm, which involves the use of partial derivatives with the intent to minimize the loss function. Hence, the main goal during the training of DNN is to find optimal weights, such that the loss function is minimized. In the present study, weights were initialized with a normal distribution with mean zero and variance 10^− 4^, and the loss function included a ridge penalization (L2) which is represented in matrix notation as follows:

$$ \mathrm{L}\left(\mathbf{y},\hat{\mathbf{o}}\right)={\left(\mathbf{y}-\hat{\mathbf{o}}\right)}^{\mathrm{t}}\left(\mathbf{y}-\hat{\mathbf{o}}\right)+\uplambda {\mathbf{w}}^{\mathrm{t}}\mathbf{w} $$

where $\mathrm{L}\left(\mathbf{y},\hat{\mathbf{o}}\right)$ is the penalized loss function, **y** is the vector of pre-corrected body weights, $\hat{\mathbf{o}}$ is vector of predicted body weights, **w** is a vector of DNN weights, and λ is the regularization parameter. Moreover, the DNN optimization was performed with the Adam algorithm, which is an extension of the stochastic gradient descent that accounts for the first and second moments of the gradients [45]. Deep neural networks were trained with a learning rate of 10^− 4^ and a mini-batch size of 256 instances.

A common challenge when modeling DNN is overfitting. Besides to the ridge penalization, we implemented DNN using two additional approaches to tackle potential overfitting: early stopping and dropout [44]. In early stopping, a part of the training set, referred to as tuning set, was used exclusively to monitor the performance of DNN. The mean square error (MSE) in the tuning set was computed for every 5 epochs, and the learning process stopped if no improvement was observed after 5 consecutive assessments of the MSE in the tuning set. The maximum number of epochs allowed to train each DNN was 1000. In the dropout approach, units from DNN are randomly removed during training in a way that in the forward propagation, units are not active, and in the backward propagation their corresponding weights are not updated. Either early stopping or dropout strategies are important approaches to improve generalization of DNN models, and thus mitigate overfitting. Another critical step of DNN is to search for the best architecture since it can drastically influence predictive performance. To address this problem, we have searched for neural networks hyperparameters by taking advantage of a parallel computing system available at University of Wisconsin-Madison through the Center for High Throughput Computing (CHTC), in which 200 different architectures of DNN were tested simultaneously. Random combinations of all hyperparameters included in the search space [i.e. number of hidden layers, number of units per layer, ridge regularization (L2), and a common dropout rate to each layer] were tested to select the best DNN model. More detailed information about the hyperparameters used in the searching process are depicted in Table 1. All DNN analyses were implemented in Python using the TensorFlow library [46].

### Assessment of predictive performance

The predictive performance of each method was assessed by splitting the data into training and testing sets, in which the chronological information of 18 maternal grandsire generations was used as a criterion to divide the entire data. The former set included 67,741 broilers related to the oldest 16 generations of maternal grandsire, while the latter set contained 9735 broilers originated from the newest two generations of maternal grandsire. This validation scheme was considered in our study because, in practice, genomic breeding values of selection candidates (younger individuals) are predicted using marker effects estimated based on the information of a reference population (older individuals). To investigate the impact of the training sample size on predictive performance, 15 subsets of the training set were created, by sub-sampling 1, 3, 5, 7, 10, 15, 20, 30, 40, 50, 60, 70, 80, 90, and 100% of the original training set size. Broilers were randomly assigned to each subset with the constraint that larger subsets contained all animals from smaller sets (i.e., 1% ⊂ 3% ⊂ … ⊂ 100%). Furthermore, a tuning set was created to implement the early stopping approach for DNN. The tuning set included 4225 broilers descendants from the most recent generation of maternal grandsire in the training set. Therefore, all predictive approaches were trained using broilers related to the oldest 15 generations of maternal grandsire, and the tuning set was used exclusively to monitor the predictive performance in DNN. Keeping an exclusive data set to fine-tune hyperparameters favors DNN compared to Bayesian regression approaches as the latter do not required parameter optimization. To make comparisons fairer, the Bayesian Ridge Regression and Bayes Cπ were also refitted by including the tuning set of the DNN in each sub-sampling of the training set. Therefore, these new models (denoted by BRR-WT and the Bayes Cπ-WT) correspond to the same BRR and Bayes Cπ models previously described, but fitted with the addition of the tuning set into each sub-sampling of the training set.

The criteria used to assess predictive performance were the prediction correlation, i.e. correlation between the pre-adjusted and the predicted body weights, and the MSEP, given by:

$$ MSEP=\frac{\sum \limits_{i=1}^{n_{test}}{\left({y}_i^{\ast }-{\hat{y}}_i\right)}^2}{n_{test}}, $$

where ${y}_i^{\ast }$ is the pre-adjusted body weight from the *i*^*th*^ broiler in the testing set, ${\overset{\frown }{y}}_i$ is the predicted genomic breeding value for body weight, and *n*_*test*_ is the number of records in the testing set. Furthermore, improvements in prediction correlation and MSEP of DNN compared with Bayesian models were assessed using the relative gain (RG), which was measured as follows:

$$ \mathrm{RG}=\frac{\left({\mathrm{r}}_1-{\mathrm{r}}_2\right)}{{\mathrm{r}}_2}\times 100, $$

where r_1_ and r_2_ are the predictive criterion (i.e. prediction correlation or MSEP) for DNN and Bayesian approaches, respectively.

The predictive bias was also investigated for each genome-enabled prediction approach as the deviation of the regression coefficient between pre-adjusted and predicted body weight from the unit. In addition, the Spearman rank correlation between predicted body weights, and the agreement on the top 10-ranked broilers were used to assess the similarity between the different genome-enabled prediction approaches.

## Data Availability

The data that support the findings of this study are available from Cobb upon reasonable request with signed confidentiality agreement contract by communicating with Rachel J. Hawken (rachel.hawken@cobb-vantress.com).

## Abbreviations

ANN: Artificial Neural Networks
Bayes Cπ-WT: Bayes Cπ fitted including the tuning set in the training set
BRR: Bayesian Ridge Regression
BRR-WT: Bayesian Ridge Regression fitted including the tuning set in the training set
CNN: Convolution neural networks
DNN: Deep neural networks
MLPs: Multilayer perceptron
MSE: Mean square error
MSEP: Mean square error of prediction
RG: Relative gain
SNP: Single nucleotide polymorphism
